# Supplementary material for: Long-Term Efficacy and Safety of Evinacumab in Patients with Homozygous Familial Hypercholesterolemia: Real-World Clinical Experience
Source: Pharmaceuticals (Basel). 2022 Nov 11;15(11):1389. doi: 10.3390/ph15111389 (PMC9698659; doi:10.3390/ph15111389)
Supplement: Supplementary file 1 [file pharmaceuticals-15-01389-s001.zip › pharmaceuticals-1981814-supplementary.pdf]

Figure S1. Percentage change from baseline in LDL-cholesterol with lipoprotein apheresis alone for individual patients on background of conventional lipid-lowering therapies

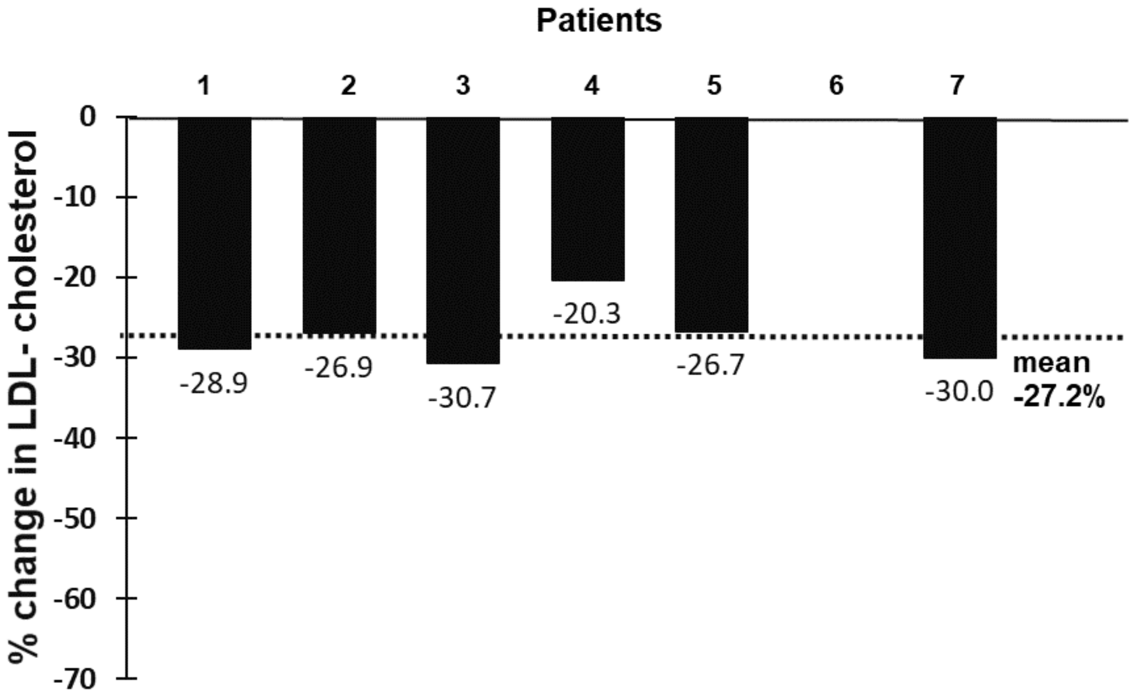

* Patient 6 did not receive lipoprotein apheresis treatment

Figure S2. Change from baseline in total cholesterol (TC) (A), non-HDL-cholesterol (B) and apoB (C) during 24-month treatment for individual patients.

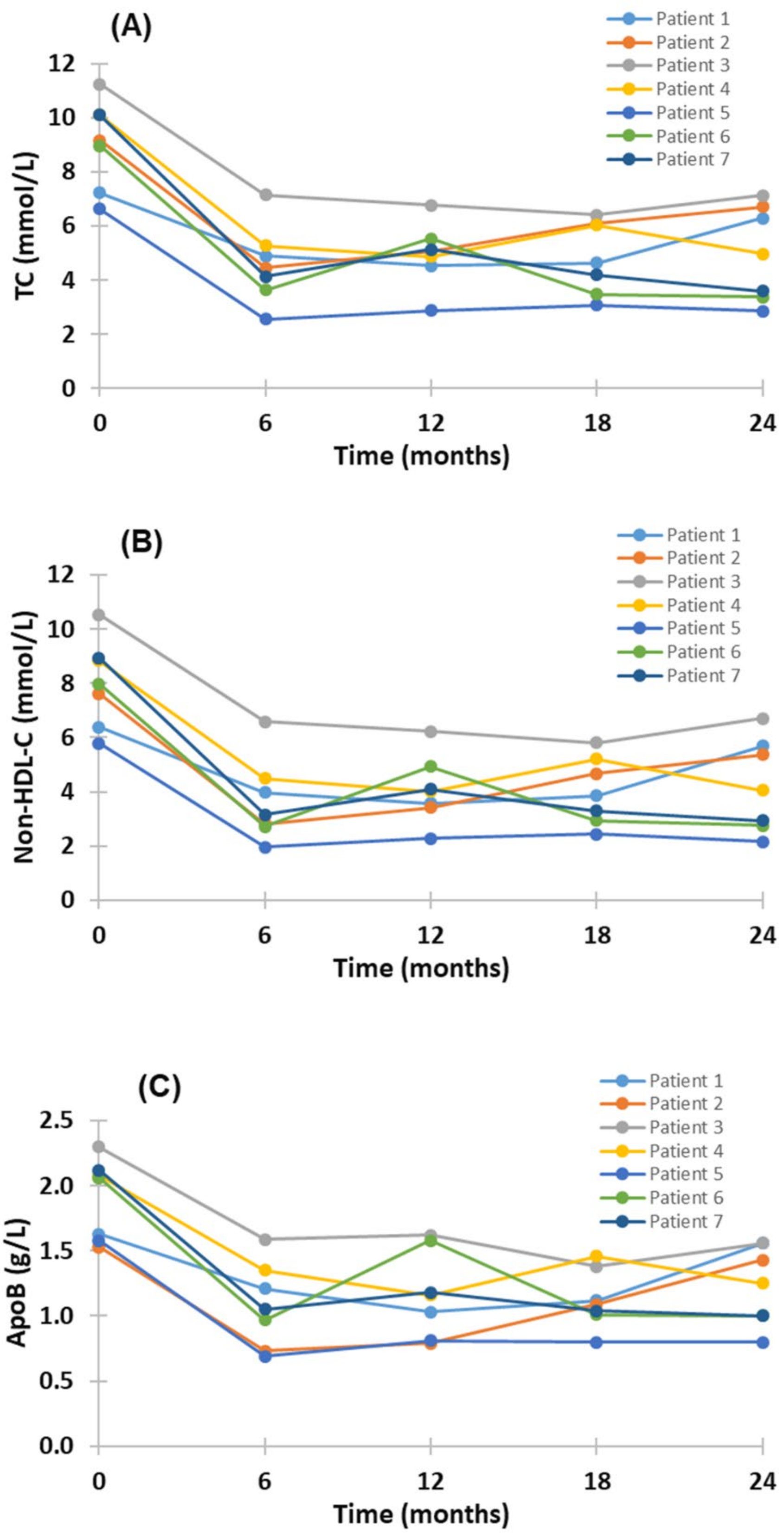

Figure S3. Change in plasma high-sensitivity C-reactive protein (hsCRP) concentration with evinacumab and lipoprotein apheresis during 24-month treatment (A) and the corresponding percentage change (B) at 24-month follow-up (B) for individual patients

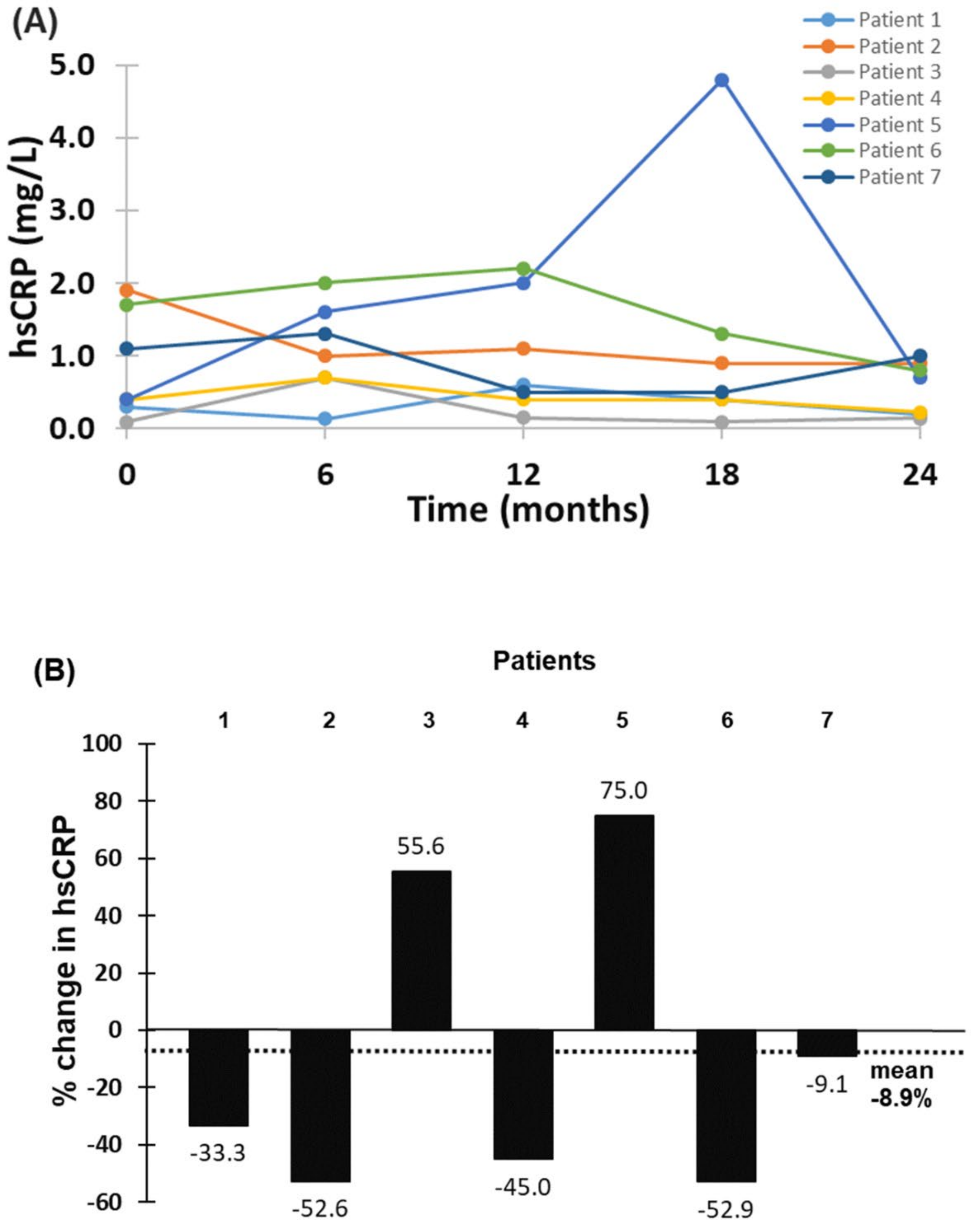

Table S1. Plasma lipid, lipoprotein and apolipoprtoeins concentrations of individual HoFH patient before and during therapy with evinacumab.

Patient No	Time	TC (mmol/L)	TG (mmol/L)	HDL-C (mmol/L)	Non-HDL-C (mmol/L)	LDL-C (mmol/L)	REM-C (mmol/L)	ApoA-I (g/L)	ApoB (g/L)	ApoC-III (mg/L)	Lp(a) (nmol/L)
1	Pre-treatment	7.2	1.79	0.85	6.38	5.5	0.84	0.97	1.63	111	17
	6 months	4.9	0.63	0.93	3.97	3.7	0.29	0.90	1.21	38	14
	12 months	4.5	0.66	0.96	3.57	3.3	0.31	0.85	1.03	37	15
	18 months	4.6	0.66	0.78	3.86	3.6	0.31	0.85	1.12	36	14
	24 months	6.3	0.77	0.60	5.69	5.3	0.35	0.77	1.56	45	12
2	Pre-treatment	9.2	1.32	1.55	7.62	7.0	0.60	1.43	1.53	NA	113
	6 months	4.5	0.32	1.63	2.82	2.7	0.15	1.26	0.73	37	86
	12 months	5.1	0.29	1.66	3.42	3.3	0.13	1.22	0.79	29	90
	18 months	6.1	0.36	1.42	4.67	4.5	0.16	1.27	1.09	30	127
	24 months	6.7	0.37	1.35	5.36	5.2	0.18	1.32	1.43	48	112
3	Pre-treatment	11.2	0.88	0.70	10.5	10.13	0.41	0.78	2.3	66	8
	6 months	7.2	0.63	0.57	6.6	6.29	0.29	0.62	1.6	24	9
	12 months	6.8	0.64	0.54	6.2	5.93	0.29	0.65	1.6	23	7
	18 months	6.4	0.52	0.62	5.8	5.57	0.23	0.71	1.4	32	9
	24 months	7.1	0.55	0.41	6.7	6.45	0.26	0.51	1.6	16	4
4	Pre-treatment	10.1	1.16	1.27	8.83	8.3	0.54	1.29	2.08	124	169
	6 months	5.3	0.53	0.80	4.48	4.3	0.23	0.89	1.35	20	156
	12 months	4.9	0.42	0.85	4.02	3.8	0.19	0.91	1.16	32	141
	18 months	6.0	0.51	0.83	5.20	5.0	0.23	1.00	1.46	27	172
	24 months	5.0	0.52	0.91	4.06	3.8	0.23	1.03	1.25	34	190
5	Pre-treatment	6.6	0.76	0.85	5.78	5.4	0.34	0.99	1.58	56	16
	6 months	2.6	0.53	0.60	1.96	1.7	0.22	0.67	0.69	<9.4	12
	12 months	2.9	0.54	0.60	2.27	2.0	0.25	0.72	0.81	<9.4	13
	18 months	3.1	0.50	0.65	2.43	2.2	0.23	0.78	0.80	<9.4	12
	24 months	2.9	0.51	0.70	2.15	1.9	0.23	0.77	0.80	15	12
6	Pre-treatment	9.0	1.86	0.98	7.98	7.1	0.86	1.16	2.06	123.3	122
	6 months	3.6	0.95	0.91	2.72	2.3	0.44	1.03	0.97	27.6	47
	12 months	5.5	1.02	0.62	4.92	4.5	0.47	0.82	1.58	21.6	53
	18 months	3.5	0.80	0.54	2.93	2.6	0.37	0.74	1.01	<9.4	66
	24 months	3.4	0.95	0.62	2.75	2.3	0.44	0.77	1.00	20.4	32
7	Pre-treatment	10.1	0.53	1.17	8.93	8.7	0.23	1.31	2.12	NA	157
	6 months	4.1	0.5	0.98	3.16	2.9	0.23	1.03	1.05	11.9	65
	12 months	5.1	0.47	1.04	4.09	3.9	0.20	1.08	1.18	23.3	77
	18 months	4.2	0.43	0.91	3.29	3.1	0.21	1.01	1.04	11.20	82
	24 months	3.6	0.41	0.67	2.93	2.8	0.18	0.75	1.00	<9.4	65
